# Gradual Acquisition of Professional Knowledge, Audit Quality and Audit Fees

**DOI:** 10.3389/fpsyg.2022.759875

**Published:** 2022-03-17

**Authors:** Gaoshuang Xu, Rui Ding, Hanxiu Cheng, Qiuhang Xing

**Affiliations:** ^1^School of Economics and Management, Nanjing University of Science and Technology, Nanjing, China; ^2^Business School, University of Shanghai for Science and Technology, Shanghai, China; ^3^Guanghua School of Management, Peking University, Beijing, China

**Keywords:** professional knowledge, auditor industry expertise, audit quality, audit fee, time factor

## Abstract

The acquisition of professional knowledge is a core issue in the formation of auditor industry expertise; however, previous literature has neglected the time required for auditors to acquire professional knowledge. We examine the audit quality and fees of audit firms in different stages of an auditor acquiring professional knowledge and find that, in the initial stage of the process of knowledge acquisition, audit quality and audit fees decrease. However, in the long run, knowledge learning has a more obvious effect on the improvement of audit quality and audit fees. Specifically, knowledge learning has a positive effect on the development of audit firms.

## Introduction

The development of audit firms is of great significance for the healthy operation of the capital market (Becker et al., 1998), and knowledge management is important for audit firms’ development. Developing a knowledge management strategy helps audit firms gain a competitive advantage in the market. Industry specialization is an important knowledge management strategy for audit firms to achieve differentiated development.

How can audit firms form industry specializations? This issue is of great concern for both practical and theoretical research (Craswell et al., 1995; Solomon et al., 1999; Gramling and Stone, 2001; Gaver and Utke, 2019). Previous theoretical studies indicate that the formation of auditors’ industry specialization needs to meet at least two key factors: first, audit firms need to occupy a large market share in a certain industry (Craswell et al., 1995). Second, auditors need to audit the same type of company multiple times to accumulate experience and knowledge, thereby forming more mature audit methods and knowledge for specific industries (Gendron et al., 2007). The first factor, the importance of market share, has become a consensus in research on auditors’ industry specialization. This is also why the extant literature adopts the industry market share of audit firms as the criteria for determining whether auditors are industry specialists. However, few studies have focused on the second factor, which we refer to as the time factor. Gaver and Utke (2019) use data from the United States audit market to find that there is no difference in audit quality between industry experts who have recently become and other non-specialist auditors. Meanwhile, only audit firms that have been industry experts for a long time will provide significantly higher audit quality. This verifies that time plays an important role in the formation of industry specialization.

We assume that ignoring the importance of time in the formation of industry expertise results in biased theoretical research conclusions. Currently, the literature on identifying audit firms generally adopts the industry market share method, which sets an industry market share threshold; all audit firms that reach this threshold are identified as industry specialists (DeFond and Zhang, 2014). The implicit assumption of using this method to identify industry specialists is that once the market share of an audit firm reaches the artificially recognized threshold, it will immediately obtain industry-specific auditing capabilities far exceeding other auditors, that is, the formation of industry expertise is “one-time” rather than “step by step.” According to the conclusions of Gaver and Utke (2019), this assumption is likely to be invalid. If this assumption is not valid, the conclusions of the literature that used this measurement will be biased.

From the perspective of the time factor, this study divides auditor industry specialists into unseasoned specialists and seasoned specialists based on the length of time the audit firm reaches the market share threshold of industry specialists. The results show that the establishment of industry specialists cannot be achieved overnight. The acquisition of professional knowledge is time-consuming. Compared with unseasoned specialists, seasoned specialists with accumulated years of experience can provide higher audit quality and charge higher audit fees. In addition, unseasoned specialists use “low-balling” to reach the market share threshold and quickly occupy the market, which results in even lower audit fees than that of non-specialist auditors. Simultaneously, the rapidly expanding market has surpassed the carrying capacity of unseasoned specialists in the short term, resulting in an insufficient supply of audit resources for audit firms, which in turn results in lower audit quality than that of non-specialist auditors. Therefore, in the short run, audit firms experience a decrease in audit quality and fees when implementing industry specialization strategies. However, in the long run, specialization strategy has a more obvious effect on the improvement of audit quality and audit fees; that is, specialization strategy has a positive effect on the development of audit firms.

There are two important dimensions for evaluating an audit firm’s development: audit quality and audit fees. Our study makes several contributions from these two dimensions. First, it re-examines the relationship between auditors’ professional expertise and audit quality from the time dimension. Cai and Xian (2007) were the first to study this important issue in China and find that auditor industry specialists have lower audit quality than non-specialist auditors, which they attribute to the lower level of development of industry expertise in Chinese audit firms. Later, Liu et al. (2010), however, use a special sample of financially fraudulent firms to test but find that auditor industry specialists can improve audit quality. Our study partially answers the reasons for the contradictory research conclusions in the literature on the Chinese capital market and provides a new perspective and evidence for research on Chinese industry expertise. Second, the literature does not consider the time factor when studying the relationship between industry expertise formation and audit fees. This study provides an important supplement to this issue, which has not been studied by Gaver and Utke (2019). For audit firms, the goal of implementing industry specialization strategies is to gain a competitive advantage and obtain excess profits, and audit fees are an important criterion for evaluating the effectiveness of the strategy. Therefore, studying the relationship between auditor industry expertise and audit fees from the perspective of time is of great significance for a comprehensive understanding of the implementation process of industry specialization strategies.

The remainder of this study is organized as follows. We first develop the hypotheses and then describe the methodology used in this study (sample selection and research design) in Section “Methodology.” Section “Results” discusses the empirical results and robustness test in Section “Robustness Tests.” The final section concludes this paper.

## Hypothesis Development

In previous literature focused on the industry expertise of auditors, the market share of audit firms in a certain industry was used as a measure of industry expertise (Behn et al., 2008; Lim and Tan, 2008; Payne, 2008; Reichelt and Wang, 2010; Fung et al., 2012). The literature has examined the relationship between auditors’ industry expertise and audit quality. Previous literature finds that audit firms with a large industry market share have deepened their understanding of clients in specific industries and have obtained audit knowledge and skills in specific industries through professional investment and repeated audits (Behn et al., 2008; Lim and Tan, 2008; Payne, 2008; Reichelt and Wang, 2010; Fung et al., 2012). This may bring many benefits to auditors and clients, including improvement in audit quality (Owhoso et al., 2002; Balsam et al., 2003; Gaver and Utke, 2019), disclosure quality of clients’ information (Dunn and Mayhew, 2004; Moroney, 2007; Reichelt and Wang, 2010), audit fees (Bae et al., 2016), and audit efficiency (Dekeyser et al., 2019). Audit firms with a larger market share will have more industry-specific knowledge and experience, be able to identify industry-specific problems more accurately, and possess a differentiated competitive advantage over other firms to charge premiums for audit fees (Bae et al., 2016). At the same time, clients are more willing to hire high-paying auditors with industry expertise because of their motivation to improve the quality of financial information disclosure (Craswell et al., 1995).

However, it is not complete to consider only the industry market share when studying auditors’ industry expertise in the past. The formation of the industry expertise of the audit firm is a gradual process, which requires at least the following two conditions. First, the audit firm needs to occupy a large market share in a certain industry to conduct repeated audits in different companies in the same industry. Second, when occupying a certain market share, auditors also need to work in this industry for a certain period, exploring, verifying, and summarizing specialized audit knowledge and methods obtained during multiple audits to form an industry-specific experience (Gaver and Utke, 2019). Therefore, we can assume that the time factor is important in the formation of industry expertise. Most extant literature uses the industry market share method to measure industry expertise, which sets a market share threshold and identifies the audit firm that reaches the threshold as an industry specialist. This method ignores the important role of time in the formation of industry expertise. When the market share of an audit firm in an industry has just reached the standard identified in theoretical research, it is impossible to immediately obtain audit knowledge and methods specific to the industry and to immediately become an industry expert indeed (Gaver and Utke, 2019).

To correct these potential problems, we consider the time factor in the empirical design and define industry specialists whose market share has just reached the prescribed threshold for one year as unseasoned specialists and industry specialists whose market share has reached the prescribed threshold for more than one year as seasoned specialists. In addition, auditors that do not meet the prescribed market share thresholds are non-specialist auditors.

We classify audit firms into three categories: seasoned specialists, unseasoned specialists, and non-specialist auditors. By comparing the audit quality and audit fees of the three types of auditors, especially the differences between unseasoned specialists and the other two types of auditors, we can gain a clearer understanding of the time factor in the formation of industry expertise.

### Differences in Audit Quality Between Different Types of Auditors

The theory of skill acquisition points out that the cultivation of skills is not an overnight process but a step-by-step process that needs to go through the verbal-cognitive, motor, and autonomous stages. It takes time for individuals to acquire and apply knowledge through extensive practice to become proficient (Fitts, 1964; Van Wijk et al., 2008). The biggest difference between unseasoned specialists and seasoned specialists is that unseasoned specialists have a short period of time to acquire new industry clients, are unable to immediately explore, verify, and summarize industry-specific audit knowledge and skills, and are not yet familiar with industry-specific business practices, internal controls, audit procedures, and other factors; therefore, there is a large gap between the quality of audit services provided by unseasoned specialists and seasoned specialists in the short term (Gaver and Utke, 2019).

Based on the above analysis, we propose the following hypothesis 1:

Hypothesis 1: The audit quality of seasoned specialists is higher than that of unseasoned ones.

However, unseasoned specialists may have the same audit capabilities as seasoned specialists. Although unseasoned specialists have just reached the market share threshold, they have also shown a gradual increase in market share over the preceding years. In the process of gradually acquiring more clients in the industry before reaching the threshold, the expertise of unseasoned specialists may have increased to a higher level. As a result, the audit quality of seasoned specialists may not be higher than that of unseasoned ones. The difference in audit quality between seasoned and unseasoned specialists remains an empirical question that needs to be tested.

Seasoned specialists have held a larger market share for many years (Gaver and Utke, 2019), have a better understanding of the industry’s clients, are aware of the risk points in industry audits, and are more capable of designing appropriate audit solutions to control audit risk (Solomon et al., 1999; Balsam et al., 2003; Romanus et al., 2008; Chi and Chin, 2011), thus providing significantly higher audit quality than non-specialist auditors.

Based on the above analysis, we propose the following hypothesis 2:

Hypothesis 2: The audit quality of seasoned specialists is higher than that of non-specialist auditors.

However, non-specialist auditors may have the same audit quality as seasoned specialists. This is because although non-specialist auditors do not have strong audit expertise, they may use other means to compensate for the lack of competence in order to survive in a highly competitive auditing market, such as by increasing audit effort to improve audit quality. Therefore, the audit quality of seasoned specialists may not be higher than that of a non-specialist auditor. The difference in audit quality between seasoned specialists and non-specialist auditors remains an empirical question to be tested.

Beginners learn the basics of skills through observation and imitation and then gradually digest and absorb knowledge through continuous practice and flexible use (Fitts, 1964; Dreyfus and Dreyfus, 2004). Compared to non-specialist auditors, unseasoned specialists have only just reached the threshold of market share, have just started their audit work with clients, and, therefore, have not yet fully accumulated and mastered their expertise and do not differ significantly from non-specialist auditors in terms of audit competency (Gaver and Utke, 2019). When facing a sudden increase in a large number of new industry clients, unseasoned specialists may face a shortage of audit resources in the short term (Bills et al., 2016), and are unable to perform effective audits of client companies, providing lower quality audit services than non-specialist auditors. Based on the above analysis, we propose the following hypothesis 3:

Hypothesis 3: The audit quality of unseasoned specialists is lower than that of non-specialist auditors.

However, as noted earlier, although unseasoned specialists have recently reached the market share threshold, industry expertise may improve as they continue to increase their market share. Consequently, the audit quality of unseasoned specialists may also be higher than that of non-specialist auditors. The difference in audit quality between unseasoned specialists and non-specialist auditors remains an empirical question to be tested.

### Differences in Audit Fees Between Different Types of Auditors

Compared with unseasoned specialists, seasoned specialists have more industry audit experience, higher audit quality, and a better reputation in the industry (Gaver and Utke, 2019), and listed companies are willing to hire seasoned industry specialists and pay higher fees to improve the quality of financial reports and convey reliable financial report information to the market (Basioudis and Francis, 2007).^1^ Brand life cycle theory points out that the establishment of a brand is not achieved overnight, but needs to go through the process of creation, stabilization, imitation, differentiation, and polarization stage (Simon, 1979). Similarly, it will take time for the reputation of a new industry specialist to build and spread. Newly promoted industry specialists have just occupied a certain market share, audit quality has not yet been improved, and their reputation in the industry has not yet been established; therefore, audit fees cannot be increased immediately, and there is a significant gap with the fees of seasoned industry experts. Based on the above analysis, we propose the following hypothesis 4:

Hypothesis 4: Audit fees of seasoned specialists are higher than those of unseasoned specialists.

However, as mentioned earlier, although unseasoned specialists have only recently reached the market share threshold, their industry expertise may be improving and their reputation may be growing as they continue to acquire new clients to reach the market share threshold. Thus, the reputation of an unseasoned specialist may not be weaker than that of a seasoned specialist and there may be no difference in audit fees that client firms are willing to pay between the two. The difference in audit fees between seasoned and unseasoned specialists remains an empirical question to be tested.

The audit quality of seasoned specialists is higher (Romanus et al., 2008; Chi and Chin, 2011), so clients are more willing to pay a premium for the audit of seasoned specialists (Basioudis and Francis, 2007). Simultaneously, seasoned specialists gradually build a reputation over time and are more recognized by listed companies (Craswell et al., 1995). Therefore, audit fees for seasoned specialists are higher than those for non-specialist auditors.

Based on the above analysis, we propose the following hypothesis 5:

Hypothesis 5: Audit fees of seasoned specialists are higher than those of non-specialist auditors.

However, although seasoned specialists have higher audit competencies than non-specialist auditors do, non-specialist auditors can compensate for the lack of competencies by, for example, increasing their audit effort, thus ensuring audit quality and maintaining their reputation. Non-industry experts who improve audit quality by increasing audit input may be equally recognized by clients and receive high audit fees. The difference in audit fees between seasoned specialists and non-specialist auditors remains an empirical question to be tested.

The reputations of audit firms affect their audit fees (Ferguson and Stokes, 2002). In addition, the establishment and dissemination of brand awareness take a certain amount of time (Simon, 1979). Unseasoned specialists have only captured a certain market share, audit quality has not yet improved, and their reputation in the industry has not yet been established. Therefore, from the client’s perspective, there is no significant difference in the quality of service and reputation between unseasoned specialists and non-specialist auditors and there is no need to pay more for unseasoned specialists. Moreover, in the face of fierce competition in the audit market, unseasoned specialists are likely to acquire new clients using low prices (DeAngelo, 1981; Ettredge and Greenberg, 1990), thus reaching the market share threshold. Therefore, emerging industry specialists may charge even lower audit fees than non-specialist auditors. Based on the above analysis, we propose the following hypothesis 6:

Hypothesis 6: The audit fees of unseasoned specialists are lower than those of non-specialist auditors.

However, both the competence and reputation of unseasoned specialists gradually accumulate as they continue to acquire new clients within the industry, and the fact that unseasoned specialists reach a market-share threshold may be an indication of the establishment of their reputation. Therefore, when an emerging industry expert reaches the market share threshold, it is likely that an unseasoned specialist has gained market recognition and can receive a higher audit fee than a non-specialist auditor. The difference in audit fees between unseasoned specialists and non-specialist auditors remains an empirical question to be tested.

## Methodology

The hypotheses proposed in this study compare the differences between seasoned specialists, unseasoned specialists, and non-specialist auditors. Therefore, we adopt the same research design as Gaver and Utke (2019) to design regression models (1) and (2) to examine the impact of industry expertise on audit quality and audit fees separately:


Ln(P/(P-1))=β+0βS1easoned+βU2nseasoned

(1) +C⁢o⁢n⁢t⁢r⁢o⁢l⁢s+ε

LAF=β+0βS1easoned+βU2nseasoned

(2) +C⁢o⁢n⁢t⁢r⁢o⁢l⁢s+ε

In Model (1), we use financial restatement to measure audit quality, which takes a value of 1 if the company restates its financial report for the year and 0 otherwise (Lobo and Zhao, 2013; Czerney et al., 2014; Guo et al., 2016; Zhang, 2019). Financial restatement is a direct measure of audit quality because the company’s financial restatement means that the auditor does not detect and correct the financial misstatement; that is, audit quality is low (DeFond and Zhang, 2014). In model (2), *LAF* is the natural logarithm of audit fees. When the dependent variable is financial restatement (*Restatement*), we adopt the logit regression model, whereas when the dependent variable is the audit fee (*LAF*), we adopt the OLS regression model.

The key independent variables are seasoned specialists (*SEASONED*) and unseasoned specialists (*UNSEASONED*). First, according to Chen et al. (2010), this study defines an industry expert (*Expert*) as if the audit firm responsible for the audit is the industry leader or the industry market share is greater than 10%, then it is 1; otherwise, it is 0. Industry market share is the sum of the logarithm of client company assets in a certain industry of the audit firm divided by the sum of the logarithm of assets of all companies in the industry in that year. Then, we distinguish seasoned specialists (*SEASONED*) from unseasoned specialists (*UNSEASONED*) according to the length of time when the market share of the audit firm reaches the threshold. *SEASONED* is defined as 1 if the audit firm is an industry expert and has been an industry expert for more than one year, and 0 otherwise. *UNSEASONED* is defined as 1 if the audit firm is an industry expert and the time of becoming an industry expert equals 1 year, otherwise, it is 0. We use the example in the Appendix to better show the way we define the variables. We assumed that all listed companies in the publicly traded manufacturing sector were audited by four audit firms (audit firms A, B, C, and D) between 2002 and 2006. We assume that 2003 was the first year in which the four audit firms mentioned above entered the manufacturing sector to conduct audits. Appendix shows the industry market share of each audit firm in each year; for example, audit firm A had an industry market share of 20% in 2003. Using audit firm C as an example, if we focus only on the market share percentage, as defined in the literature, audit firm C is considered a non-specialist auditor if it has less than 10% market share in 2002 and 2003. Audit firm C is considered an industry expert if it reached the 10% market share threshold in 2004 and subsequent years. In our study, we consider not only the audit firm’s market share but also the length of time since the audit firm reached the market share threshold. Since 2004 was the first year in which audit firm C reached 10% market share, audit firm C was identified as an unseasoned specialist at this time. In subsequent years, audit firm C maintained a market share of 10% or more and was recognized as a *SEASONED* audit firm in subsequent years. Similarly, we classify other auditing firms. Audit firms are classified into three categories: seasoned specialists, unseasoned specialists, and non-specialist auditors.

In this way, *SEASONED* and *UNSEASONED* divide the samples into three categories, where *SEASONED* equals 1 representing companies audited by seasoned specialists and *UNSEASONED* equals 1 representing companies audited by unseasoned specialists. When *SEASONED* and *UNSEASONED* equal 0 at the same time, benchmark companies are audited by non-specialist auditors. We expect that the coefficient of *SEASONED* in Model (1) is significantly negative, which means that the audit quality of seasoned specialists is significantly higher than that of non-specialist auditors, and the coefficient of *UNSEASONED* is significantly positive, which means that the audit quality of unseasoned specialists is lower than that of non-specialist auditors. In Model (2), we assume that the coefficient of *SEASONED* is significantly positive, which means that the audit fees of seasoned specialists are significantly higher than those of non-specialist auditors; the coefficient of *UNSEASONED* is significantly negative, which means that the audit fees of unseasoned specialists are lower than those of non-specialist auditors.

Hypothesis 1 and 4 assume significant differences in audit fees and audit quality between seasoned and unseasoned specialists. We exclude the observations audited by non-specialist auditors, include only *SEASONED* in the model, and judge the difference between seasoned specialists and unseasoned specialists by examining the significance of the coefficient before *SEASONED*. We use the following models (3) and (4) to examine H1 and H4:


(3) Restatement=β+0βS1easoned+Controls+ε

(4) LAF=β+0βS1easoned+Controls+ε

We expect that the coefficient of *SEASONED* in Model (3) is significantly negative, which means that the audit quality of seasoned specialists is significantly higher than that of unseasoned specialists. In Model (4), we assume that the coefficient of *SEASONED* is significantly positive, which means that the audit fees of seasoned specialists are significantly higher than those of unseasoned specialists.

We also include the following control variables in the regression model (1) -- (4): company size (*Size*), which is the natural logarithm of the company’s total assets; company’s asset-liability ratio (*LEV*), which represents the ratio of total liabilities to total assets; company profitability (*ROA*), which is the ratio of net profit to total assets; cash flow status (*CFO*), which equals the current operating cash flow divided by total assets; company’s listing time (*Age*), which is the number of years the company is listed; the nature of the company’s property rights (*SOE*), it equals 1 when the company is a state-owned firm and is 0 otherwise; the tenure of the audit firm (*Tenure*), which is the number of consecutive years the client company has been audited by its current audit firm;^2^ the nature of audit firms (*IBig4*), which equals 1 if the audit is performed by one of the Big Four audit firms (PwC, E&Y, KPMG, or Deloitte) and is 0 otherwise. Industry and year fixed^3^ (Gow et al., 2010). The central limit theorem states that when there are sufficient observations, the distribution of the variables converges to a normal distribution. Owing to the large amount of data used in this study, problems that do not satisfy the normal distribution should have less impact on the conclusions of this study. Nevertheless, we still perform a logarithmic treatment of audit fees to ensure that they conform to a normal distribution as much as possible. To avoid the influence of outliers, we winsorize all continuous variables at the 1 and 99% levels.

We first select the 2003–2018 listed companies in the non-financial industry from CSMAR as the initial sample, then exclude 3,355 samples with missing audit fee data, exclude one sample with missing data for computing industry expertise, and finally exclude 379 observations with missing data for control variables. The final sample comprised 32,389 company-year observations. Table 1A presents the sample-selection process.

**TABLE 1:** Sample description.

**(A) Sample selection**			
Firm-year observations of non-financial listing firms from 2003 to 2018	36,124		
*Less:*			
Firm-years with missing data of audit fees	(3,355)		
Firm-years with missing data of industry specialization	(1)		
Firm-years with missing data of control variables	(379)		
Total	32,389		
**(B) Distribution by year**			

**Year**	**Frequency**	**Percentage (%)**	

2003	1,207	3.73	
2004	1,235	3.81	
2005	1,183	3.65	
2006	1,161	3.58	
2007	1,185	3.66	
2008	1,351	4.17	
2009	1,521	4.70	
2010	1,743	5.38	
2011	2,009	6.20	
2012	2,383	7.36	
2013	2,414	7.45	
2014	2,529	7.81	
2015	2,705	8.35	
2016	2,981	9.20	
2017	3,352	10.35	
2018	3,430	10.59	
Total	32,389	100	
**(C) Distribution by industry**			

**Year**	**Frequency**	**Percentage (%)**	

Agriculture, forestry, animal husbandry, and fishery	470	1.45	
Mining	984	3.04	
Manufacturing	16,552	51.10	
Utilities	1,350	4.17	
Construction	852	2.63	
Wholesale and retail	1,154	3.56	
Information tech.	4,772	14.73	
Real estate	2,002	6.18	
Leasing and business services	1,826	5.64	
Health and social work	1,625	5.02	
Culture, physical education, and recreation	486	1.50	
Others	316	0.98	
Total	32,389	100	
**(D) Distribution of restatement observations across audit firms**			

	**Other audit firm**	**Unseasoned specialists**	**Seasoned specialists**

Number of non-financial restatement observations	23,639	372	6,942
Number of financial restatement observations	1,214	26	139

Table 1B presents the sample distribution by year. The number of observations has increased from 1,207 (3.73%) in 2003 to 3,430 (10.59%) in 2018. The year-on-year growth trend of the observations is in line with the development of China’s capital market. Table 1C shows the distribution of the sample by industry. The largest number of observations in the manufacturing industry is 16,552, accounting for 51.10% of the total sample, consistent with the current situation in China’s capital market. Table 1D presents the distribution of restatement observations across audit firms.

## Results

### Descriptive Statistics

Table 2A presents the descriptive statistics. The average value of financial restatement (*Restatement*) is 0.043, indicating that 4.3% of the sample has financial restatements. The mean value of *SEASONED* was 0.219, indicating that 21.9% of the samples were audited by seasoned specialists, and the mean value of *UNSEASONED* was 0.012, indicating that only 1.2% of the samples were audited by unseasoned specialists. The descriptive statistics of the other variables are similar to those in the literature. Table 2B presents the correlation coefficient matrix. The correlation coefficients between most of the variables were low, indicating that the results of this study were less affected by the problem of multicollinearity.

**TABLE 2:** Descriptive statistics and correlation matrix.

(A) Descriptive statistics												
**Variable**	**Obs**	**Mean**	***SD***	**Min**	**P25**	**Median**	**P75**	**Max**				
Restatement	32,389	0.043	0.202	0.000	0.000	0.000	0.000	1.000				
LAF	32,389	13.546	0.729	12.206	13.039	13.459	13.911	16.167				
SEASONED	32,389	0.219	0.413	0.000	0.000	0.000	0.000	1.000				
UNSEASONED	32,389	0.012	0.110	0.000	0.000	0.000	0.000	1.000				
SIZE	32,389	21.830	1.296	19.027	20.907	21.692	22.584	25.768				
LEV	32,389	0.457	0.228	0.051	0.283	0.449	0.613	1.249				
ROA	32,389	0.034	0.071	−0.342	0.013	0.035	0.065	0.205				
CFO	32,389	0.044	0.077	−0.203	0.004	0.044	0.088	0.260				
AGE	32,389	10.003	6.527	0.000	4.000	9.000	15.000	25.000				
SOE	32,389	0.427	0.495	0.000	0.000	0.000	1.000	1.000				
TENURE	32,389	4.225	3.187	1.000	2.000	3.000	6.000	16.000				
IBig4	32,389	0.055	0.229	0.000	0.000	0.000	0.000	1.000				

**(B) Correlation matrix**												

**Variable**	**(1)**	**(2)**	**(3)**	**(4)**	**(5)**	**(6)**	**(7)**	**(8)**	**(9)**	**(10)**	**(11)**	**(12)**

(1) Restatement	1											
(2) LAF	−0.084***	1										
(3) SEASONED	−0.060***	0.121***	1									
(4) UNSEASONED	0.013**	−0.017***	−0.059***	1								
(5) SIZE	−0.066***	0.751***	0.043***	0.004	1							
(6) LEV	0.099***	0.186***	−0.090***	−0.001	0.276***	1						
(7) ROA	−0.114***	0.027***	0.058***	0.006	0.079***	−0.422***	1					
(8) CFO	−0.046***	0.048***	0.020***	0.005	0.065***	−0.159***	0.331***	1				
(9) AGE	−0.002	0.274***	−0.038***	−0.003	0.309***	0.324***	−0.181***	−0.054***	1			
(10) SOE	0.029***	0.129***	−0.129***	0.010*	0.286***	0.236***	−0.073***	0.047***	0.339***	1		
(11) Tenure	−0.034***	0.187***	−0.017***	−0.104***	0.197***	0.085***	−0.067***	0.019***	0.363***	0.085***	1	
(12) IBig4	−0.025***	0.433***	−0.099***	−0.018***	0.330***	0.055***	0.054***	0.085***	0.054***	0.136***	0.063***	1

***, ** and * denote significance at the 1%, 5%, and 10% level, respectively.

### Regression Results

Table 3 shows the regression results for audit quality and audit fees for seasoned and unseasoned specialists. The dependent variables in columns (1) and (3) are financial restatements (*Restatement*) and the dependent variables in columns (2) and (4) are audit fees (*LAF*). To test for differences between seasoned specialists and non-specialist auditors, and the differences between unseasoned specialists and non-specialist auditors, we use the full sample in the regressions in Columns (1) and (2). To test for differences between seasoned specialists and unseasoned specialists, we exclude observations audited by non-specialist auditors and the results are presented in Columns (3) and (4). To avoid multicollinearity, we calculated the variance inflation factor (VIF) value of the main variables, which showed that none of the VIF values were greater than 10.

**TABLE 3:** Industry specialization, audit quality, and audit fees.

Dependent variable	(1) Restatement	(2) LAF	(3) Restatement	(4) LAF
	Full sample		Exclude observations audited by non-specialist auditors	
SEASONED	−0.366***	0.108***	−0.794***	0.126***
	(−3.30)	(9.57)	(−2.77)	(5.81)
UNSEASONED	0.425**	−0.045**		
	(2.02)	(−2.21)		
SIZE	−0.007	0.347***	0.057	0.358***
	(−0.18)	(50.18)	(0.69)	(30.31)
LEV	0.744***	0.138***	1.262***	0.173***
	(3.80)	(4.69)	(2.67)	(3.24)
ROA	−2.914***	−0.325***	−2.940***	−0.145
	(−6.77)	(−5.44)	(−2.89)	(−1.16)
CFO	−1.584***	0.106**	−2.496**	0.163*
	(−3.89)	(2.21)	(−2.21)	(1.80)
AGE	0.026***	0.004***	0.046***	0.004**
	(2.97)	(3.77)	(2.66)	(2.11)
SOE	−0.138	−0.060***	−1.104***	−0.023
	(−1.64)	(−4.28)	(−4.48)	(−0.83)
TENURE	−0.050***	0.002	−0.120***	0.010***
	(−3.65)	(0.99)	(−2.99)	(2.67)
IBig4	−0.611***	0.777***	−0.372	0.786***
	(−2.86)	(20.78)	(−0.31)	(4.71)
Cons	−1.403*	5.667***	−2.370	5.357***
	(−1.74)	(39.92)	(−1.35)	(22.00)
Industry fixed effects	YES	YES	YES	YES
Year fixed effects	YES	YES	YES	YES
*N* (firm-years)	32389	32389	7479	7479
Adj. *R*^2^ (pseudo *R*^2^)	0.126	0.673	0.145	0.622

Standard errors are robust to heteroscedasticity and clustered at the firm level. T-statistics are in parentheses. ***, **, and * denote significance at the 1, 5, and 10% level, respectively.

Column (3) of Table 3 shows the regression results of H1. In Column (3), the coefficient of *SEASONED* is significantly negative (−0.794, *z* = −2.77), indicating that the audit quality of seasoned specialists is significantly higher than that of unseasoned specialists.

Column (1) of Table 3 shows the regression results of H2. From the results in the first column, we find that the coefficient of *SEASONED* is significantly negative (−0.366, *z* = −3.30), indicating that compared with non-specialist auditors, client companies audited by seasoned specialists are less likely to have financial restatements, that is, seasoned specialists have higher audit quality. Thus, Hypothesis 2 is verified.

Column (1) of Table 3 shows the regression results of H3. From the results in the first column, we find that the coefficient of *UNSEASONED* is significantly positive (0.425, *z* = 2.02), indicating that compared with non-specialist auditors, client companies audited by unseasoned specialists are more likely to have financial restatements, that is, the audit quality of unseasoned specialists is lower. Thus, Hypothesis 3 is verified.

Column (4) of Table 3 shows the regression results of H4. In Column (4), the coefficient of *SEASONED* is significantly positive (0.126, *t* = 5.81), indicating that audit fees of seasoned specialists are significantly higher than those of unseasoned specialists.

Column (2) of Table 3 shows the regression results of H5. From the results in the second column, it can be seen that the coefficient of *SEASONED* is significantly positive (0.108, *t* = 9.57), indicating that compared with non-specialist auditors, the audit fees of client companies audited by seasoned specialists are higher. Thus, Hypothesis 5 is verified.

Column (2) of Table 3 shows the regression results of H6. From the results in the second column, it can be seen that the coefficient of *UNSEASONED* was significantly negative (−0.045, *t* = −2.21), indicating that, compared with non-specialist auditors, unseasoned specialists charge lower fees. Thus, Hypothesis 6 is verified.

## Robustness Tests

### Propensity Score Matching

The differences between clients of industry-specialist auditors and clients of non-specialist auditors may be large, which may constitute an alternative interpretation of the results. Therefore, we use propensity score matching to minimize the differences in various observable variables between the sample of non-specialist auditors and the sample of industry-specialist auditors (Austin, 2011; Lennox et al., 2012; Beck and Lisowsky, 2014; Shipman et al., 2017). First, we calculated the propensity score matching value according to Model (5):


(5) Expert=β+0Controls+ε

where *Expert* indicates whether the company is audited by industry specialists; if it is, it is 1; otherwise, it is 0. We control for the same variables as in model (1) in model (5). Because *Expert* is a dummy variable, we conduct a logit regression model. Table 4 shows the results of propensity score matching, among which Panel A shows the matching model. We use the matching model to match 7479 observations audited by industry-specialist auditors with their corresponding control group samples. Among them, six observations of industry-specialist auditors do not meet the common support hypothesis and could not be matched with appropriate observations of non-specialist auditors; therefore, 7473 observations of industry-specialist auditors remained. The nearest neighbor matching method without replacement is used to match the observation of non-specialist auditors for each observation of industry-specialist auditors, and finally, 14,946 samples after matching are obtained. Panel B shows the comparison result of the mean value of the control variables between the sample of industry-specialist auditors and the sample of non-specialist auditors after matching. As shown in Table 4B, there is no significant difference between the sample of industry-specialist auditors and the sample of non-specialist auditors after matching, indicating that the matching effect is better. Panel C shows the regression results after matching, and the main conclusion does not change.

**TABLE 4:** Propensity score matching.

(A) Matching model				

**Dependent variable**			**Expert**	
SIZE			0.119***	
			(8.11)	
LEV			−0.101	
			(−1.19)	
ROA			0.577**	
			(2.21)	
CFO			0.854***	
			(4.05)	
AGE			−0.019***	
			(−7.12)	
SOE			−0.185***	
			(−5.13)	
TENURE			−0.044***	
			(−8.63)	
IBig4			−1.856***	
			(−16.84)	
Cons			−2.426***	
			(−7.22)	
Industry fixed effects			YES	
Year fixed effects			YES	
*N* (firm-years)			32389	
Pseudo *R*^2^			0.129	

**(B) Comparison result of the mean value of the control variables after matching**				

**Variable**	**Expert = 1**	**Expert = 0**	***t***	***p* > |*t*|**

SIZE	21.933	21.949	−0.77	0.441
LEV	0.420	0.420	−0.11	0.91
ROA	0.041	0.040	0.74	0.457
CFO	0.047	0.047	−0.12	0.905
AGE	9.555	9.673	−1.04	0.297
SOE	0.315	0.323	−1.05	0.293
TENURE	3.975	3.944	0.66	0.511
IBig4	0.013	0.015	−0.91	0.363

**(C) Regression result after matching**				

**Dependent variable**		**(1) Restatement**		**(2) LAF**

SEASONED		−0.381***		0.098***
		(−2.95)		(8.40)
UNSEASONED		0.424*		−0.052**
		(1.83)		(−2.51)
SIZE		0.099*		0.349***
		(1.68)		(42.18)
LEV		0.533		0.121***
		(1.57)		(3.34)
ROA		−3.869***		−0.294***
		(−4.66)		(−3.35)
CFO		−1.945**		0.205***
		(−2.43)		(3.16)
AGE		0.029**		0.005***
		(2.41)		(3.76)
SOE	−0.542***	−0.048***
	(−3.49)	(−2.59)
TENURE	−0.091***	0.002
	(−3.59)	(1.06)
IBig4	−2.061**	0.848***
	(−2.11)	(10.58)
Cons	−3.665***	5.654***
	(−2.92)	(32.58)
Industry fixed effects	YES	YES
Year fixed effects	YES	YES
*N* (firm-years)	14946	14946
Adj. *R*^2^ (Pseudo *R*^2^)	0.091	0.608

Standard errors are robust to heteroscedasticity and clustered at the firm level. T-statistics are in parentheses. ***, **, and * denote significance at the 1, 5, and 10% level, respectively.

### Entropy Balance Matching

DeFond et al. (2017) pointed out that although the propensity score matching method can alleviate the estimation problems caused by the large difference between two different groups, the results of this method are greatly affected by the model setting and choice of specific methods. Therefore, we used another entropy balancing method commonly used in international journals for robustness testing (Hainmueller, 2012; Haislip et al., 2017; Wilde, 2017; Glendening et al., 2019; McMullin and Schonberger, 2020). This method adjusts the weight of each sample in one group such that there is no significant difference between the two groups for each control variable. Entropy-balance matching has several advantages. First, the nearest neighbor matching used in the propensity score matching method must exclude unmatched group samples, which will cause a large loss of original information. Compared with the PSM method, entropy balance assigns different weights to each observation to preserve the most valuable information when preprocessing the data and maintain the efficiency of subsequent analysis (Hainmueller, 2012; Wilde, 2017; McMullin and Schonberger, 2020). Second, the propensity score matching method can choose to replace or not, choose a different matching radius, and choose one-to-one matching or one-to-many matching. Therefore, the different choices may have a greater impact on the final results. However, there is no choice of specific methods in entropy matching; therefore, there is no research design difference in the results. We formed a new sample by adjusting the weight of each observation in the sample of non-specialist auditors. Table 5 presents the results of the regression of the samples after entropy balance matching. It can be seen from Table 5, the main conclusions have not changed, indicating that the conclusions of this article are relatively robust.

**TABLE 5:** Entropy balance matching.

Dependent variable	(1) Restatement	(2) LAF
SEASONED	−0.424***	0.106***
	(−3.68)	(9.37)
UNSEASONED	0.407*	−0.035*
	(1.86)	(−1.73)
SIZE	0.017	0.358***
	(0.38)	(41.38)
LEV	0.781***	0.123***
	(3.24)	(3.46)
ROA	−3.541***	−0.289***
	(−6.70)	(−3.65)
CFO	−1.917***	0.172***
	(−3.51)	(2.95)
AGE	0.030***	0.004***
	(2.86)	(3.21)
SOE	−0.366***	−0.046***
	(−3.58)	(−2.64)
TENURE	−0.069***	0.003
	(−4.49)	(1.39)
IBig4	−0.755**	0.786***
	(−2.01)	(10.28)
Cons	−1.788*	5.432***
	(−1.89)	(30.87)
Industry fixed effects	YES	YES
Year fixed effects	YES	YES
*N* (firm-years)	32389	32389
Adj. *R*^2^ (Pseudo *R*^2^)	0.118	0.627

Standard errors are robust to heteroscedasticity and clustered at the firm level. T-statistics are in parentheses. ***, **, and * denote significance at the 1, 5, and 10% level, respectively.

## Conclusion

Our study uses listed companies from 2003 to 2018 as a sample to examine the impact of auditors’ industry expertise on audit quality and audit fees from the perspective of time factor. We divide industry specialists into unseasoned specialists and seasoned specialists based on the length of time the audit firm’s market share in a certain industry has reached a prescribed threshold. This shows that compared with seasoned specialists, the audit quality and audit fees of unseasoned specialists are lower. This shows that the formation of industry specialization cannot be accomplished overnight, and only the market share reaching the threshold set by industry specialists is not sufficient to become an industry specialist. Only after years of accumulation can audit firms truly gain industry expertise, provide higher audit quality, and be recognized by client companies to charge higher fees. Our study also finds that compared with non-specialist auditors, unseasoned specialists have lower audit quality and lower audit fees. It proves that audit firms will take the “low-balling” strategy to quickly occupy the market. Thus, industry specialists will initially charge even lower fees than non-specialist auditors. At the same time, the rapidly expanding market has surpassed the carrying capacity of audit firms in the short term, resulting in an insufficient supply of resources for audit firms, which in turn leads to a decline in audit quality. These results remain after various robustness tests.

The implementation method and effect of the auditor industry’s professionalization strategy is a fundamental issue in the field of audit theory research, as well as an important issue for the audit firm. Our study uses Chinese data to provide a more accurate and detailed answer to this question. The empirical results of this study have important implications for regulatory agencies in formulating policies for the selection of audit firms’ competitive strategies. On the one hand, the Chinese Institute of Certified Public Accountants should further strengthen and implement the policy of making audit firms larger and stronger, especially guiding audit firms to develop their industry expertise, improve audit quality, charge premiums, and promote the healthy development of the industry. However, both regulatory authorities and audit firms should pay attention to the long-term negative impact of “low-balling” on audit fees at the initial stage of the implementation of the industry specialization strategy and the short-term negative impact of the rapid expansion of market share on audit quality.

## Data Availability Statement

Publicly available datasets were analyzed in this study. This data can be found here: https://www.gtarsc.com/.

## Author Contributions

GX and RD contributed to the study design, developed the theoretical framework, were responsible for data interpretation, and wrote the first draft of the manuscript. QX was responsible for the design and development of the data analysis. HC collected the data. QX and HC reviewed the manuscript. All authors approved the manuscript.

## Conflict of Interest

The authors declare that the research was conducted in the absence of any commercial or financial relationships that could be construed as a potential conflict of interest.

## Publisher’s Note

All claims expressed in this article are solely those of the authors and do not necessarily represent those of their affiliated organizations, or those of the publisher, the editors and the reviewers. Any product that may be evaluated in this article, or claim that may be made by its manufacturer, is not guaranteed or endorsed by the publisher.

## Footnotes

1 Audit services are different from other products in that the quality of audit services is difficult to be perceived directly. Although clients are willing to pay more for high quality audit services, clients choose and pay audit firms based on their reputation (not actual audit quality), and there may be cases where clients pay higher audit fees but do not receive higher quality audit services.

2 Audit tenure in this context refers to the length of time the audit firm has provided audit services to the current client firm on a continuous basis and is only a reflection of the auditor’s knowledge of the client firm. We do not use this audit tenure when determining whether an industry expert is a seasoned industry expert, but rather the length of time the auditor has achieved and maintained a certain market share, reflecting the audit firm’s knowledge of a particular industry, not just a single client firm. Audit firms can provide audit services to individual companies for a longer period of time, but are not industry experts or are unable to consistently maintain a leading position in the industry to become seasoned experts.

3 We also use the cluster-robust standard errors to mitigate heteroscedasticity (White, 1980), the results do not change.

## Appendix

**Table:** Examples of industry expert definitions.

	Audit firm A	Audit firm B	Audit firm C	Audit firm D
2002	0% (Non-specialist)	0% (Non-specialist)	0% (Non-specialist)	0% (Non-specialist)
2003	20% (UNSEASONED)	70% (UNSEASONED)	5% (Non-specialist)	5% (Non-specialist)
2004	20% (SEASONED)	70% (SEASONED)	10% (UNSEASONED)	0% (Non-specialist)
2005	5% (Non-specialist)	70% (SEASONED)	15% (SEASONED)	0% (Non-specialist)
2006	5% (Non-specialist)	65% (SEASONED)	20% (SEASONED)	0% (Non-specialist)
